# Rapid, Effective, and Versatile Extraction of Gluten in Food with Application on Different Immunological Methods

**DOI:** 10.3390/foods10030652

**Published:** 2021-03-19

**Authors:** Verónica Segura, Jacobo Díaz, Ángela Ruiz-Carnicer, Alba Muñoz-Suano, Carolina Carrillo-Carrión, Carolina Sousa, Ángel Cebolla, Isabel Comino

**Affiliations:** 1 Department of Microbiology and Parasitology, Faculty of Pharmacy, University of Seville, 41012 Seville, Spain; vsegura@us.es (V.S.); acarnicer@us.es (Á.R.-C.); csoumar@us.es (C.S.); 2 Clinical Analysis Service, Hospital Universitario INGESA, 51003 Ceuta, Spain; jacobodp@gmail.com; 3 Biomedal S.L., 41900 Seville, Spain; amunozsuano@gmail.com (A.M.-S.); carrillocarrion.carolina@gmail.com (C.C.-C.); acebolla@biomedal.com (Á.C.)

**Keywords:** celiac disease, gluten proteins, gluten-free diet, gluten extraction solution

## Abstract

One of the main concerns in gluten analysis is to achieve efficient extraction of gluten proteins. Conventional ethanol-based extraction solutions are inefficient and, because of this, it is necessary to use reducing agents or acids for proper solubilization. The extraction recommended by CODEX Standard 118-1979 (revised 2008) utilizes Cocktail solution (patent WO 02/092633 A1). However, it is harmful with a disgusting odor and is not compatible with some immunological techniques. Here, the versatility and extraction capacity of a new Universal Gluten Extraction Solution (UGES) (patent ES 2 392 412 A1) were evaluated using different methodological conditions, food matrices, and various immunological methods. UGES includes safer compounds for both the user and the environment, and it displayed similar extraction efficiency to that of the extraction method recommended for sandwich enzyme-linked immunosorbent assay (ELISA). The extraction time was significantly reduced from 100 to 40 min, depending on the type of the sample. Furthermore, unlike the currently used solution, UGES is compatible with competitive ELISA.

## 1. Introduction

Gluten is a complex mixture of prolamin and glutelin storage proteins, which is present in certain cereals such as wheat, barley, rye, oats, and their derivates. Prolamins mainly comprise monomeric proteins that are insoluble in water and salt solutions but soluble in aqueous alcohols [1]. Prolamins contribute to the cohesiveness and extensibility of gluten. Glutelins are polymerized by interchain disulfide bonds and are insoluble in water, salt solutions, and aqueous alcohol. Glutelins play a role in the maintenance of the elasticity and strength of the gluten [2,3,4]. These common dietary proteins have unusual biochemical properties that include a high abundance of glutamine and proline residues, which render them resistant to degradation by gastrointestinal proteases [5,6].

The consumption of gluten proteins drives adverse immune reactions in predisposed individuals who suffer from wheat allergy, non-celiac gluten sensitivity (NCGS), dermatitis herpetiformis, gluten ataxia, or celiac disease (CD) [7]. CD is one of the most frequent hypersensitivities, with a global seroprevalence of 1.4% [8]. This disease is triggered by the presence of peptides generated by the fragmentation of gluten proteins that are not digested by human proteases. These peptides cross the intestinal barrier and cause an aberrant immune reaction. This results in villous atrophy accompanied by intestinal manifestations, such as abdominal pain, bloating, nausea, vomiting, diarrhea, and extraintestinal manifestations that include ataxia, anemia, and osteoporosis [9,10,11,12,13,14,15].

For decades, compliance to a strict gluten-free diet (GFD) has been accepted as the only effective treatment for CD [16] and, more recently, for patients with other gluten-related disorders [17,18]. Adherence to a GFD can be difficult, due to lack of awareness, cross-contamination of foods, and inadequate labeling and testing mechanisms [13,19,20,21,22]. The lack of other treatments for gluten-associated disorders makes sensitive and low-cost analytical methods necessary for the quantification of gluten content in all kinds of harmful samples, including foods, beverages, cosmetics, and medicines [23,24,25,26]. 

The Codex Alimentarius and Food and Drug Administration recommend that the quantification of gluten in food should be based on sensitive and specific methods with a detection limit of ≤20 ppm [27,28]. However, the GFD Certification Organization and Celiac Support Association require lower limits of ≤10 ppm ≤5 ppm gluten, respectively [26]. Gluten analytical methods should be validated and calibrated against certified reference materials. In addition, the methods should use an antibody that reacts with the cereal protein fractions toxic for those with CD and that does not cross-react with other food components, to prevent under-reporting or over-reporting gluten [18,29,30].

One of the major concerns in gluten analysis is to achieve the efficient extraction of gluten proteins from simple samples (e.g., raw materials and unprocessed products) and complex samples (e.g., heat-processed products or samples with a complex matrix composition). Conventional ethanol-based solutions inefficiently extract gluten from processed foods because, during the processing of same foods, the generated peptide profile is very dependent on numerous parameters (e.g., ingredients, time, temperature, type of fermenting organisms, or enzymes used). As a result, some gliadins/glutelins are not in their native form. To solve this problem, the use of reducing agents, acids, or enzymes is necessary to disrupt the disulfide bonds before their solubilization in alcohol solutions during the extraction procedure [31,32,33,34].

The current recommended official method is an extraction procedure with Cocktail solution. However, this solution contains guanidine hydrochloride (2 M) and 2-mercaptoethanol (250 mM) which are harmful to users, while 2-mercaptoethanol has an unpleasant odor. In addition, Cocktail solution is not compatible with some immunological assays that rely on the direct contact of an antibody with the sample extract, such as the competitive enzyme-linked immunosorbent assay (ELISA) [34,35,36]. Therefore, the search for gluten extraction solutions that are efficient in extracting proteins, compatible with different immunological methods, and safe for the user is an important aim.

Based on the development of compatible solutions, the Universal Gluten Extraction Solution (UGES) is proposed as a new gluten extraction solution. UGES features solubilizing and antiseptic agents in an ethanol solution, with no components that are toxic to the user. Here, we evaluated the efficiency, versatility, and extraction capacity of UGES using different methodological conditions and food matrices of differing complexity. Furthermore, we assessed the compatibility of this solution with various immunological methods, such as ELISA and lateral flow immunoassay (LFIA), compared with traditional extraction solutions.

## 2. Materials and Methods

### 2.1. Reagents

All chemicals were of analytical grade or higher. All aqueous solvents and solutions were prepared with double-distilled water. Prolamin Working Group (PWG) gliadin provided by the Working Group on Prolamin Analysis and Toxicity was used as the reference material. Gliadin solution (2 mg/mL) was prepared in 60% (*v*/*v*) ethanol.

### 2.2. Samples

A wide variety of commercial samples, labeled and not labeled as being gluten-free, were selected and purchased in supermarkets. Solid samples were thoroughly ground to a fine powder or mixture using an electric blender (John Oster Manufacturing Company, Mexico, Mexico). Liquid samples were homogenized by vigorous vortexing using a Licuos apparatus (Deltalab, Barcelona, Spain). The products were grouped into two food categories: heat-processed or complex composition samples and non-heat-processed samples. Information about the nutritional composition and ingredients was directly collected from the food manufacturer’s website and the product label. Rice flour was used as the negative control, and wheat flour was used as the positive control.

#### 2.2.1. Digestion of the Gliadin Standard

To reproduce gastric digestion in vitro, PWG gliadin was suspended in 0.03 N hydrochloric acid and incubated at 37 °C for 10 min with stirring. Pepsin (Sigma Aldrich, St Louis, MO, USA) was added and incubated at 37 °C for 60 min with stirring. The reaction was then stopped by incubating gliadin in a dry bath at 95 °C for 5 min to inactivate the enzyme. Gastric digests were adjusted to pH 6.0 with sodium phosphate buffer and subjected to simulated duodenal digestion by sequential addition of bovine pancreatic trypsin (Sigma Aldrich, St Louis, MO, USA) and type II bovine pancreatic-α-chymotrypsin (Sigma Aldrich, St Louis, MO, USA) at 37 °C for 30 min. The reaction was then stopped by introducing PWG gliadin in a dry bath at 95 °C for 5 min [37].

#### 2.2.2. Spiked Samples

Different commercial samples labeled as being gluten-free were spiked with PWG gliadin or digested PWG gliadin to check for a possible matrix effect that could interfere with the analysis and to evaluate the recovery. For each matrix, four samples were analyzed (gluten level: 0, 10, 20, and 40 ppm). The sample (0.5 g) in a 50 mL tube was spiked with gliadin diluted in 60% ethanol, adjusting the concentration of gliadin for each sample. The samples were incubated at 4 °C until analysis. The percentage gluten recovery (R) in foods was calculated from the average measured (M) and spiked (S) level using the equation R = (M/S) × 100.

### 2.3. Gluten Extraction Procedures

#### 2.3.1. Aqueous Ethanol Extraction

Samples (1 g) were weighed and transferred to individual polypropylene tubes (Jet Biofil, Elgin, IL, USA). Ten milliliters of 60% (*v*/*v*) ethanol were added, and the tubes were incubated in a rotary shaker for 1 h at room temperature. Each suspension was centrifuged at 2500× *g* for 10 min, and each supernatant was collected. In the case of RIDASCREEN Gliadin competitive (R-Biopharm AG, Darmstadt, Germany), an ethanol solution (60%) containing 10% liquid fish gelatin was required for the extraction of samples containing polyphenols. These samples included beer, malt, and hops. 

#### 2.3.2. Cocktail Extraction Solution

Samples (0.25 g) were weighed and individually transferred to propylene tubes. They were extracted with an extraction solution containing 2-mercaptoethanol as a reducing agent and guanidine hydrochloride as a chaotropic agent (Cocktail solution R7006, patent WO 02/092633 A1, R-Biopharm AG, Darmstadt, Germany). Briefly, 2.5 mL of the extraction solution was added to each sample, followed by incubation at 50 °C in a water bath for 40 min. Then, 7.5 mL of 80% (*v*/*v*) ethanol was added and incubated in a rotary shaker for 1 h at room temperature. Lastly, each suspension was centrifuged at 2500× *g* for 10 min, and the supernatant was collected. 

#### 2.3.3. UGES Extraction

UGES is based on a hydroalcoholic solution (less than 40%) and arginine as key components to promote gluten solubility (patent ES 2 392 412 A1).

Samples (0.5 g) were weighed and individually transferred to propylene tubes. They were then extracted with 5 mL of UGES (Hygiena, Seville, Spain). For samples containing polyphenols (including tannins) and cosmetics containing antioxidants, 0.5 g of the special polyphenol additive (Hygiena, Seville, Spain) was added. Each suspension was centrifuged at 2500× *g* for 10 min, and the supernatant was transferred to a clean tube.

### 2.4. Techniques Employed

#### 2.4.1. Enzyme-Linked Immunosorbent Assays (ELISAs)

Gluten in foods was determined using the RIDASCREEN^®^ FAST Gliadin (R-Biopharm) and RIDASCREEN^®^ Gliadin competitive (R-Biopharm) ELISA kits. Both assays utilize the R5 monoclonal antibody (moAb) [38,39]. All samples were analyzed according to the manufacturer’s instructions.

#### 2.4.2. Sodium Dodecyl Sulfate Polyacrylamide Gel Electrophoresis (SDS-PAGE) and G12/A1 Western Blotting

SDS-PAGE and immunoblotting were performed under standard conditions. Samples were diluted in SDS-PAGE denaturing buffer (62.5 mM Tris–HCl pH 6.8, 5% 2-mercaptoethanol, 2% SDS, 0.001% bromophenol blue, and 10% glycerol) [40,41,42]. Proteins resolved by 12.5% SDS-PAGE were transferred to a polyvinylidene difluoride (PVDF) membrane. The membranes were incubated with G12 or A1 moAbs o/n at 4 °C. After washing, anti-mouse immunoglobulin G (IgG) alkaline phosphatase conjugated antibody (Sigma Aldrich) was added and incubated for 1 h at room temperature. 

#### 2.4.3. Lateral Flow Immunochromatographic Assays (LFIA)

The LFIA GlutenTox Stick was used to develop the new analytical method (Hygiena, Seville, Spain). These sticks are based on the G12 and A1 moAbs. The sticks were calibrated against PWG gliadin. The food samples were extracted with 10 mL of UGES per g of food. After extraction, the samples were diluted 1:10 in dilution solution. The test sticks were dipped into the dilution solution (100 μL) in a well of a microtiter plate. The results were determined quantitatively after 30 min. The GlutenTox^®^ Reader (Hygiena, Seville, Spain) was used for quantification by continuous scanning of the sticks using an optical detector. 

### 2.5. Statistical Analysis

Data analyses was performed using SPSS 25.0 for Windows (IBM SPSS Statistics, Armonk, NY: IBM Corp). Scatter diagrams were used as graphic indicators of the commutability between the values generated by each pair of extraction solutions. The equation of the line was calculated using the nonparametric Passing–Bablok regression [43,44,45]. This method is a statistical procedure that allows valuable estimation of analytical method agreement and possible systematic bias between them. The material was deemed commutable if the ratio obtained for a reference material using the two methods was consistent with the prediction interval (ISO, 2009). The Lin concordance correlation coefficient is the concordance (rLin) between a new test or measurement and a gold standard test or measurement. This coefficient combines a precision measure, represented by the Pearson’s correlation coefficient (rPerason), with an accuracy measure, represented by the bias correction coefficient (Cb). The McBrige scale was used to more stringently qualify the strength of the agreement. For continuous variables, the scale was almost perfect for values >0.99, substantial for values of 0.95 to 0.99, moderate for values of 0.90 to 0.95, and poor for values <0.90. Differences between groups were statistically compared by the median using the Wilcoxon test.

## 3. Results and Discussion

### 3.1. Characterization of UGES Protocol

During the processing of some foods, the heat treatment of cooked and baked products leads to the formation of protein aggregates and gluten-derived peptides in insoluble matrices. This results in a heterogeneous mixture with a nonuniform distribution of gluten, which makes analyses even more difficult. It is necessary to optimize the extraction systems to produce the complete release and recovery of both prolamins and glutelins [46,47]. The time and temperature of incubation during the extraction process are two critical factors to consider [48]. 

**Figure 1:**
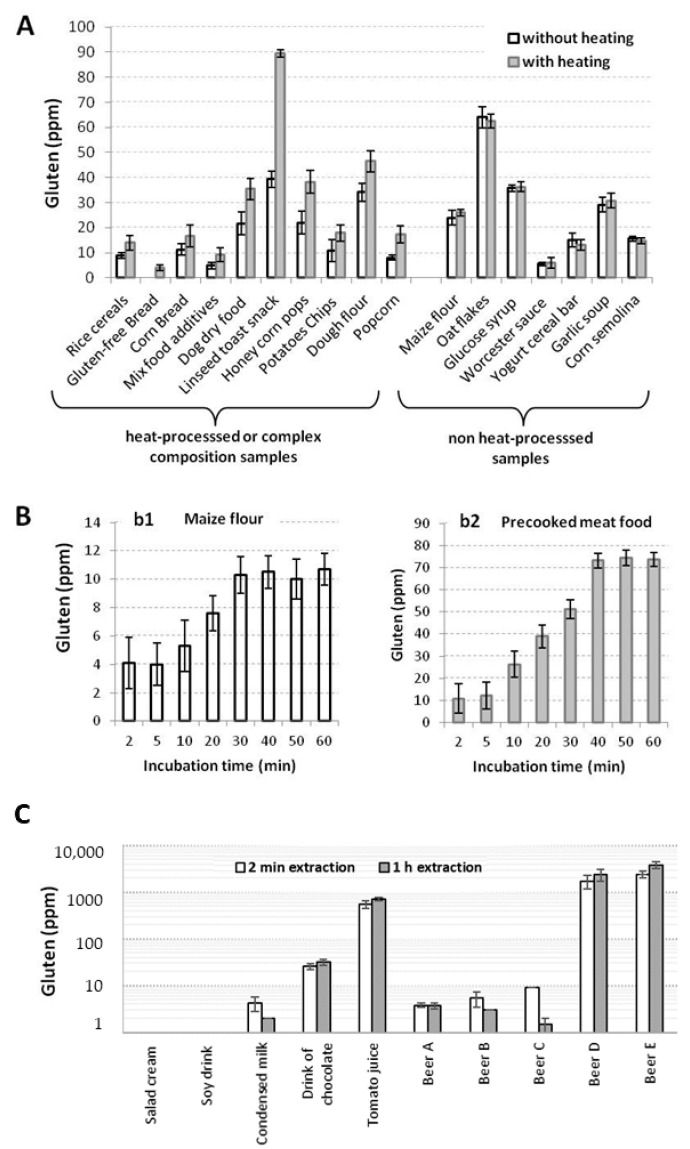
Optimization of time and temperature in the Universal Gluten Extraction Solution (UGES) protocol. (**A**) Effect of temperature in different types of samples. (**B**) Effect of time in simple and complex solid samples. (**C**) Effect of extraction time in liquid samples without suspended solids. The experiments were performed in duplicate, and the mean ± standard deviation (SD) is shown.

In this work, the effect of temperature on UGES was evaluated (Figure 1). Several samples were extracted at two conditions: incubation in wheel agitation at room temperature and incubation in a water bath at 50 °C. In both cases, the time was fixed at 1 h, and the amount of extracted gluten was quantified by sandwich ELISA (Figure 1A). Unheated processed food or simple samples (maize flour, oat flakes) did not need heat to achieve the complete extraction of gluten. In contrast, heat treatment was needed for complete extraction in samples with a complex composition (mixture food additives) and for heat-processed samples (corn bread).

The influence of different incubation periods (2 min to 1 h) was studied. Maize flour (b1) and a precooked meat food (b2) were selected to optimize the extraction time. The b1 example was extracted by rotation at room temperature. The b2 example was heat-extracted. A minimum of 30 to 40 min was necessary to achieve the complete gluten extraction for both samples (Figure 1B). According to these results, the extraction time was fixed at 40 min for solids samples or containing polyphenol, tannins, or antioxidants, regardless of the use of heating in the extraction.

Several additional assays were carried out with liquid samples without solids in suspension, such as milk, juices, organic drinks, beers, and soft drinks, to evaluate if the extraction time for these samples could be reduced. The results obtained by competitive ELISA indicated a reduction in the extraction time of these samples because they were satisfactory with only 2 min of agitation compared to the extraction carried out in solid samples (40 min) (Figure 1C).

These findings indicated that the optimum extraction procedure depends largely on the type of sample. However, the advantage of UGES is its adaptability to different types of samples with slight modifications to the protocol, as described in Table 1.

### 3.2. UGES Extraction Effectiveness Assessed by Sandwich ELISA 

#### 3.2.1. Determination of Gluten in Commercial Food Samples

Having characterized UGES using different matrices, we next evaluated the application of UGES to the analysis of different commercial foods, which were labeled and not labeled as being gluten-free, by comparison to standard extraction procedure using 60% ethanol or Cocktail solution.

UGES was highly efficient for simple and complex matrices, even with thermal processing. The gluten amount extracted with UGES was always higher (between 1.1- and 5.3-fold increase) than that extracted with 60% ethanol (Table 2). In addition, we found five food samples with a gluten content <20 ppm using 60% ethanol solution but >20 ppm gluten using UGES. The greater increases with UGES compared with 60% ethanol corresponded to heat-processed samples, such as corn bread, linseed toast snack food, and chips. Gluten proteins contain intermolecular bonds, which allow them to form a network when cooked or baked [2]. The results obtained demonstrated that the use of reducing and disaggregating agents is, thus, necessary to break disulfide bonds during the extraction procedure. Ethanol–water solutions used as extractants solubilize monomeric prolamins in non-processed food like flour. However, their efficiency is low when processed food is analyzed because protein aggregation occurs due to disulfide bond formation [34,49,50].

Additionally, 93 commercial food samples, labeled and not labeled as being gluten-free, were analyzed using UGES and Cocktail solution (Table 3). Gluten was undetectable, less than the limit of quantification (<LOQ) in 51 of the samples by both extraction methods (data no shown). No significant differences in the gluten content of the food were evident using UGES and Cocktail solution; the yield factors found were 0.5 to 1.7 times. However, the “chips a” sample contained <20 ppm gluten using Cocktail solution and >20 ppm using UGES. This result is very important because individuals with CD can only ingest products with a gluten content <20 ppm.

A statistical evaluation of the extraction process was performed by comparing UGES versus ethanol and UGES versus Cocktail solution. Passing–Bablok linear regression analysis with a prediction interval of 95% was calculated to describe the commutability of the results. Lin’s concordance correlation coefficient (r_Lin_) was determined using a precision measure (r_Pearson_) and an accuracy measure (C_b_). Lastly, the differences between groups were statistically compared using the Wilcoxon test.

**Figure 2:**
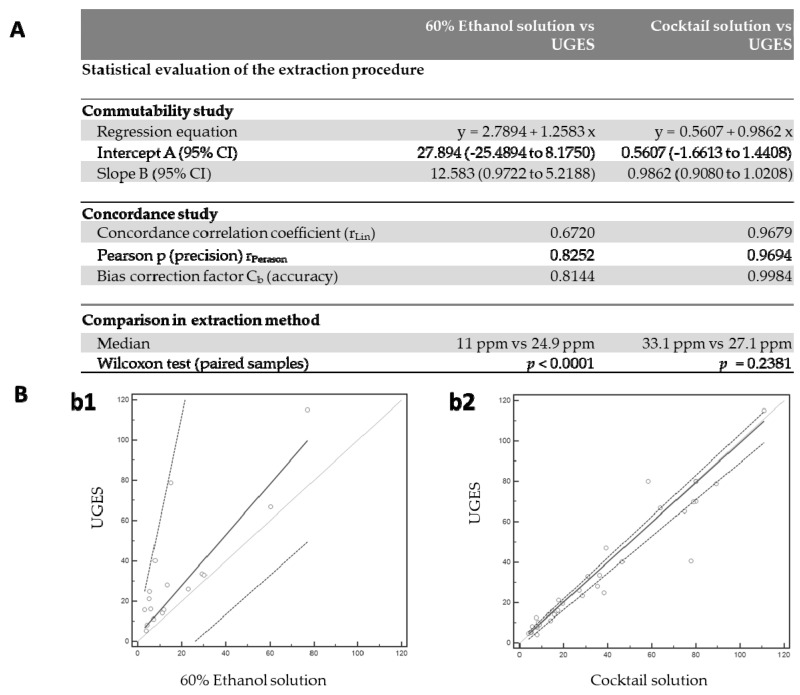
Statistical evaluation of the extraction procedure. (**A**) Comparison of agreement between pairs of assays for results of the food samples by sandwich ELISA using different extraction solutions different. Statistical analysis was performed by the Wilcoxon test. The *p*-values are given in the figure. (**B**) Scatter diagram with regression line and concordance bands for regression line; (**b1**) representation of the 60% ethanol solution versus Universal Gluten Extraction Solution (UGES); (**b2**) representation of the Cocktail solution versus UGES. The solid lines represent the regression line drawn for the food samples, and the dashed lines represent the 95% prediction interval.

The statistical analysis of the gluten concentrations obtained by UGES and 60% ethanol showed that the data were not commutable (Figure 2B(b1)), since the precision and accuracy were low (r_Pearson_ = 0.8252, C_b_ = 0.8144). This indicated a poor agreement strength, according to the McBrige scale (<0.90), with r_Lin_ = 0.6720 (Figure 2A). In addition, these results showed that UGES had a better extraction capacity than 60% ethanol solution, since there was a significant difference in the results according to the Wilcoxon test (*p* < 0.0001) (Figure 2A). However, UGES and Cocktail solution were commutable according to Passing–Bablok regression (Figure 2B(b2)) with high precision and accuracy coefficients (r_Pearson_ = 0.9694, C_b_ = 0.9984) (Figure 2A). Lin’s correlation concordance coefficient (r_Lin_) was 0.9679, indicating substantial agreement strength according to the McBrige scale (>0.95). Furthermore, no significant difference was found (*p* = 0.2381) according to the Wilcoxon test, indicating that Cocktail solution could be replaced by UGES.

#### 3.2.2. Recovery of Gliadins in Spiked Food Samples

**Figure 3:**
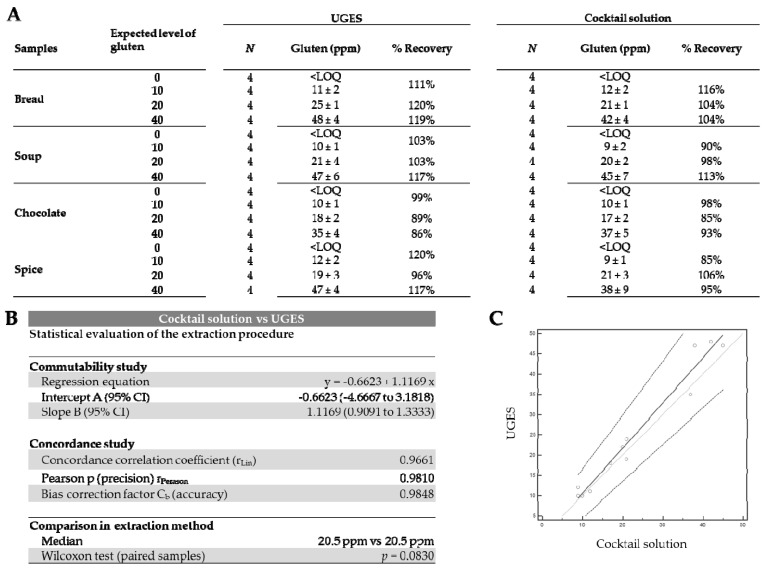
Analysis of spiked samples by sandwich ELISA after extraction with Universal Gluten Extraction Solution (UGES) and Cocktail solutions. (**A**) Recovery of the different levels of gluten provided in the food samples. Results are expressed as ppm of gluten (mean ± SD) and percentage of gliadin recovery (R). *N* = number of analyses. (**B**) Comparison of agreement between pairs of assays for results of the food samples by sandwich ELISA with different extraction solutions. (**C**) Scatter diagram with regression line and concordance bands for regression line, indicative of commutability. <LOQ, less than the limit of quantification.

An important approach in the analysis of gluten is to use an extraction system that leads to a recovery close to 100% to ensure that the products suitable for consumption by people with gluten-related disorders are truly free of gluten. To check this, commercial samples labeled as gluten-free (bread, soup, chocolate, and spices) were spiked with a PWG gliadin extract to produce gluten concentrations of 10, 20, or 40 ppm gluten (Figure 3A). Each spiked experiment was performed four times. These samples were extracted with UGES and Cocktail solution, and the extracted amount of gluten was quantified by sandwich ELISA. 

The Association of Official Agricultural Chemists (AOAC) has established the optimal recovery percentage for a spiked sample as 80% to 120% [51]. According to this criterion, the recovery of all samples was satisfactory with both methods. All non-spiked samples were below the LOQ. 

For the samples spiked with 10, 20, and 40 ppm gluten, the recoveries obtained with UGES were 99% to 120%, 89% to 120%, and 86% to 119%, respectively (Figure 3A). Sandwich ELISA combined with UGES extraction reduced the assay time; hence, the extraction of samples was reduced to 40 min compared with Cocktail solution. Passing–Bablok regression showed a low dispersion in the gluten concentrations obtained by both methods, indicating that the results were commutable (Figure 3C). A high precision and accuracy were evident (r_Pearson_ = 0.9810, C_b_ = 0.9848). Lin’s concordance correlation coefficient (r_Lin_ = 0.9661) was indicative of a substantial agreement strength according to the McBrige scale (>0.95). (Figure 3B). These results indicated that Cocktail solution could be replaced by UGES without any significant difference (*p* = 0.08330) in the amount of gluten extracted (Figure 3B).

### 3.3. UGES Extraction Effectiveness Assessed by Competitive ELISA 

During the manufacture of many foods, some processes include thermal and enzymatic reactions that can lead to gluten protein hydrolysis [52,53,54]. As it is not always possible to know with certainty, prior to the analysis, which food may contain gluten in a hydrolyzed form, it is very important to use a technique that guarantees a correct analysis. Sandwich ELISA uses two antibodies that bind to different sites on the antigen. Thus, gluten peptides with few amino acids and a single antibody binding site cannot be detected with this detection system, and the gluten content would probably be less than the actual value. However, competitive ELISA accurately quantifies of both intact and fragmented gluten because it uses only one antibody and requires only one epitope. 

**Figure 4:**
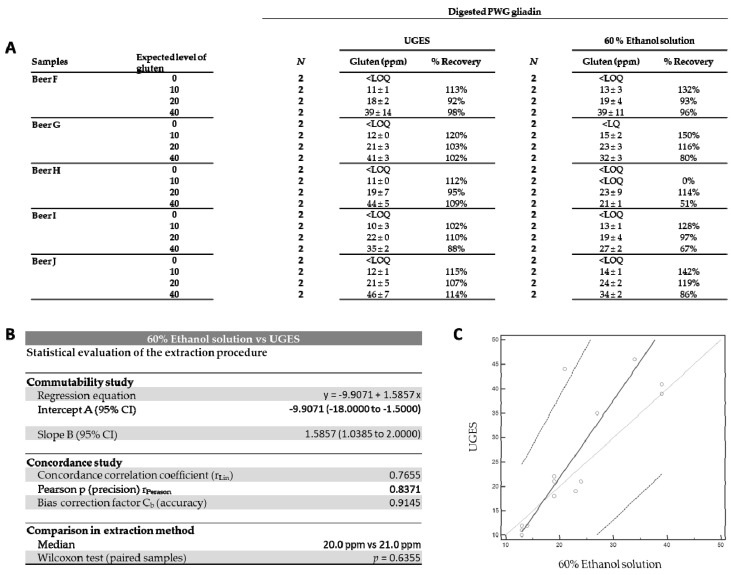
Analysis of spiked samples by competitive ELISA after extraction with Universal Gluten Extraction Solution (UGES) and 60% ethanol solutions. (**A**) Recovery of the different levels of gluten provided in the food samples. Results are expressed as ppm of gluten (mean ± SD) and percentage of gliadin recovery (R). *N* = number of analyses. (**B**) Statistical evaluation of the extraction procedure. (**C**) Scatter diagram with regression line and concordance bands for regression line. <LOQ, less than the limit of quantification; PWG, Prolamin Working Group.

To evaluate the versatility of UGES in other types of ELISA formats, we analyzed different hydrolyzed commercial gluten-free foods by spiked them with different known amounts of gluten, followed by quantification using competitive ELISA. These samples were spiked both with PWG gliadin and digested PWG gliadin in different levels (10, 20, and 40 ppm gluten) to simulate the presence of both intact gluten proteins and small peptides with a low number of epitopes. Extraction was performed with UGES, 60% ethanol, and Cocktail solutions. Before the spiking, gluten was undetectable in all samples. The recovery with Cocktail solution was between 178% and 530% in all samples; therefore, there was an overestimation of the actual gluten content (data not shown). As other authors have described [54], Cocktail solution is not compatible in assays in which the antibody comes into direct contact with the sample extract, because of the interfering effect of reducing agents (2-mercaptoethanol) in the immunoassays [34]. This component can denature the antibody [49,55]. When we used a 60% ethanol solution, we found interference with the analysis, possibly due to the effect of food matrices in the competitive ELISA. An overestimated recovery of 128% to 150% was obtained in some beers that contained a low concentration of gluten (10 ppm). In contrast, an underestimated recovery of 51% to 67% was obtained in some beers containing a high concentration of gluten (40 ppm). The recovery with 60% ethanol was 0% in the “beer H” sample spiked with 10 ppm. However, when the samples were extracted with UGES, a recovery of 80% to 120% was obtained in all the spiked samples (Figure 4A). The statistical results showed poor agreement strength when comparing the 60% ethanol solution and UGES (r_Lin_ = 0.7655, r_Pearson_ = 0.8371, C_b_ = 0.9145). However, no significant differences were found by Wilcoxon test (*p* = 0.6355) (Figure 4B). This indicated commutability at low gluten concentrations but non-commutability at higher gluten concentrations (Figure 4C).

### 3.4. UGES Extraction Using other Immunological Methods

#### 3.4.1. Western Blot

Western blot provides a qualitative analysis of proteins and, therefore, is useful for the confirmation of gluten content in foods in a manner that avoids false-positive or false-negative results [50]. Proteins separated in one-dimensional SDS-PAGE are electrotransferred onto a PVDF membrane where proteins are adsorbed. Specific antibody is added, such as G12 or A1 moAbs. These moAbs are able to recognize with great sensitivity peptides (other than the 33-mer peptide) immunotoxic for patients with CD. The sensitivity and epitope preferences of these antibodies were found to be useful for detecting the potential toxicity of food for patients with gluten-related pathologies [38,39].

**Figure 5:**
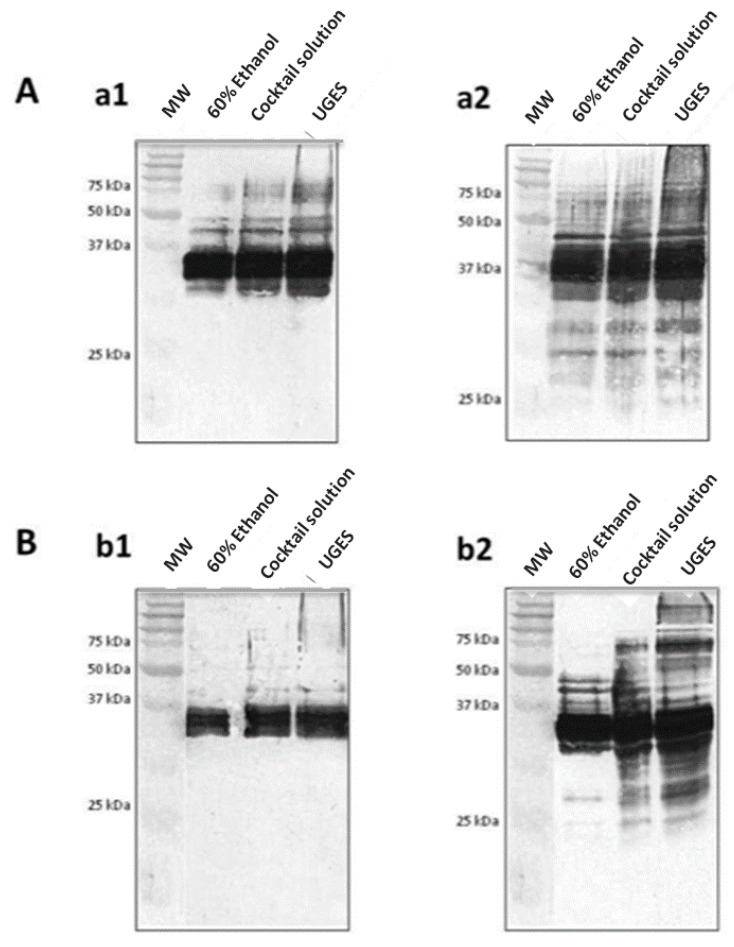
Western blot analysis of gluten using different extraction procedures. (**A**) The efficiency in the extractability of Prolamin Working Group (PWG) gliadins with (**a1**) G12 monoclonal antibody (moAb) and (**a2**) A1 moAb. (**B**) The efficiency in the extractability of gluten from two commercial samples: (**b1**) biscuits and (**b2**) wheat flour (*Triticum durum*). MW, molecular weight marker.

To prove the efficiency of the Western blot combined with UGES extraction system, we used PWG gliadin as the reference material. After extraction with 60% ethanol, most of the bands were obtained between 25 and 50 kDa with G12 (Figure 5A(a1)) and A1 (Figure 5A(a2)) moAbs. In contrast, extraction with Cocktail and UGES led to an increase in the number and intensity of bands in comparison with 60% ethanol extraction. The bands between 25 and 50 kDa (corresponding to α-, β-, and Ω-gliadins) and bands at 50–75 kDa (corresponding to α-gliadin) were significantly more intense regardless of the antibody used [56,57,58]. However, Western blot is not a quantitative method, and transfer to the membrane may vary from lane to lane.

The efficiency in the extractability of gluten in two commercial samples, one of which was heat-processed (biscuits) and the other not (wheat flour), with the different extraction procedures was examined (Figure 5B). The same amount of protein was loaded into well for the different extractions. The biggest differences were for durum wheat flour. The extraction with UGES provided bands of higher molecular weight (50–100 kDa) and lower molecular weight (37–25 kDa) in comparison with 60% ethanol extraction and an increase in the intensity of these bands with respect to the samples extracted with Cocktail solution (Figure 5B). Similar results were obtained for the A1 moAb Western blot.

#### 3.4.2. Lateral Flow Immunochromatographic Assays 

LFIA is a screening method for the detection of gluten in raw or cooked foods. The assay is rapid, highly sensitive, and feasible in a routine laboratory [25]. Therefore, we tested the efficiency of UGES combined with LFIA in gluten-free samples spiked with 20 ppm gluten (Table 4). The gluten recovery was close to 100% (92–114%) in all the matrices assayed, irrespective of the extraction procedure carried out according to the type of sample. The data proved that combining the UGES extraction with LFIA is an optimum method for confirming gluten content in different samples.

## 4. Conclusions

UGES is an effective extraction solution for gluten in a wide range of samples, including heat-treated and hydrolyzed foods. It can be considered as a universal solution compatible with various immunological methods such as Western blot, sandwich and competitive ELISA, and LFIA. This solution has better extractability than the conventional 60% ethanol solution and is at least as efficient as the sandwich ELISA Cocktail solution. On the contrary, unlike the extraction Cocktail, UGES can be successfully applied in combination with competitive ELISA. The findings highlight the versatility of UGES. The reduced extraction time and safety of the solution are compelling features supporting the routine use of UGES for gluten extraction in all kinds of consumer products. 

## Figures and Tables

**Table 1:** Scheme of the steps in the extraction procedure with UGES depending on the type of sample. RT, room temperature.

		Extraction Protocol				
Type of Sample		Amount of Sample	Additives	UGES Volume	Procedure	
Liquid	Without solids in suspension	1 mL		9 mL	Shake 1–2 min	
Solid	Non-heat-processed and simple composition	1 g		10 mL	Incubate 40 min at RT (wheel agitator)	Centrifuge 10 min at 2500× *g*
Solid	Heat-processed or complex composition	1 g		10 mL	Incubate 40 min at 50 °C (water bath)	Centrifuge 10 min at 2500× *g*
Solid or liquid	Containing polyphenols, tannins, or antioxidants	1 g	1.0 g gelatin 0.4 g PVP	10 mL	Incubate 40 min at 50 °C (water bath)	Centrifuge 10 min at 2500× *g*

PVP, polyvinylpyrrolidone.

**Table 2:** Gluten content in ppm (mg/kg) of different samples by sandwich ELISA with 60% ethanol and Universal Gluten Extraction Solution (UGES).

		Gluten (ppm)	
	Food Sample	60% Ethanol Solution	UGES *
Non-heat-processed samples	Maize flour	23	26.0 (×1)
	Corn semolina	11.7	15.7 (×1.3)
	Oat flakes	60.3	66.8 (×1.1)
	Glucose syrup	29.3	33.7 (×1.2)
	Worcester sauce	4	5.2 (×1.3)
	Yogurt cereal bar	11	14 (×1.3)
	Garlic soup	30.2	33 (×1.1)
Heat-processed or complex composition samples	Rice cereals	7	11 (×1.6)
	Gluten-free-bread	<3	4.6 (×1.5)
	Corn bread	3.2	15.7 (×4.9)
	Gluten-free breadcrumbs	4.3	8.2 (×1.9)
	Mixture food additives	<3	5.2 (×1.7)
	Spelt cracker	77.2	115.2 (×1.5)
	Dog dry food	13.3	28.2 (×2.1)
	Linseed toast snack	14.9	78.8 (×5.3)
	Honey corn pops	5.5	24.9 (×4.5)
	Chips a	5.3	21.1 (×4.0)
	Dough flour	7.9	40.2 (×5.1)
	Popcorn	6	16.1 (×2.7)

* Values in brackets are the yield factor compared to that with 60% ethanol solution and UGES.

**Table 3:** Gluten content in ppm (mg/kg) of different samples by sandwich ELISA with Cocktail solution and Universal Gluten Extraction Solution (UGES).

		Gluten (ppm)	
	Food Sample	Cocktail Solution	UGES *
Non-heat-processed samples	Maize flour	27	26.0 (×1.0)
	Corn semolina	14.8	15.7 (×1.1)
	Oat flakes	64	66.8 (×1.0)
	Glucose syrup	36.3	33.7 (×0.9)
	Worcester sauce	5.3	5.2 (×1.0)
	Yogurt cereal bar	13	14 (×1.1)
	Garlic soup	30.8	33.0 (×1.1)
Heat-processed or complex composition samples	Rice cereals	14	11 (×0.8)
	Gluten-free-bread	3.9	4.6 (×1.2)
	Corn bread	16.7	15.7 (×0.9)
	Gluten-free breadcrumbs	5.6	8.2 (×1.5)
	Mixture food additives	4.9	5.2 (×1.1)
	Spelt cracker	111	115.2 (×1.0)
	Dog dry food	35.4	28.2 (×0.8)
	Linseed toast snack	89.4	78.8 (×0.9)
	Honey corn pops	38.3	24.9 (×0.7)
	Chips a	17.8	21.1 (×1.2)
	Dough flour	46.5	40.2 (×0.9)
	Popcorn	17.4	16.1 (×0.9)
	Appetizers a	74.8	65.2 (×0.9)
	Appetizers b	80.0	70.1 (×0.9)
	Chips b	28.5	23.5 (×0.8)
	Cookies a	7.9	7.7 (×1.0)
	Cookies b	9.0	9.2 (×1.0)
	Corn arepa	7.8	4.0 (×0.5)
	Fishmeal	19.6	19.5 (×1.0)
	Infusions a	58.4	80.0 (×1.4)
	Infusions b	80.0	80.0 (×1.0)
	Lentil grains	77.7	40.6 (×0.7)
	Meat preparation	7.5	12.5 (×1.7)
	Pea flour	7.6	8.3 (×1.1)
	Plate of lentils	15.7	14.1 (x0.9)
	Quinoa flour	80.0	80.0 (×1.0)
	Rice paper	8.5	10.2 (×1.2)
	Rice preparation	80.0	80.0 (×1.0)
	Spice a	80.0	80.0 (×1.0)
	Spice b	78.9	69.8 (×0.5)
	Spice c	39.3	47.1 (×1.2)
	Sweet a	80.0	80.0 (×1.0)
	Sweet b	80.0	80.0 (×1.0)
	Sweet c	80.0	80.0 (×1.0)
	Sweet d	80.0	80.0 (×1.0)

* Values in brackets are the yield factor compared to that with Cocktail solutions and UGES.

**Table 4:** Gluten recovery in commercial samples labeled gluten-free and spiked using lateral flow immunochromatographic assay (LFIA).

		Gluten (ppm)		
Samples	Extraction Procedure	Added	Found	% Recovery
Maize flour	UGES without heating	20	22.7	114
Meat	UGES with heating	20	19.1	96
Toothpaste	UGES with heating	20	20.7	104
Body cream	UGES with additives + heating	20	18.3	92
